# Assessment of Gestational Diabetes and Associated Risk Factors and Outcomes in the Pacific Island Nation of Palau

**DOI:** 10.1007/s10995-017-2313-0

**Published:** 2017-07-26

**Authors:** Mindy S. Sugiyama, Haley L. Cash, Christine Roseveare, Rumi Reklai, Kliu Basilius, Sherilynn Madraisau

**Affiliations:** 1 Bureau of Public Health, Ministry of Health, Koror, Palau; 2 Pacific Island Health Officers Association, Honolulu, HI USA; 3 Regional Public Health Service, Koror, Palau

**Keywords:** Gestational diabetes mellitus, Diabetes, Pregnancy, Neonatal death, Obesity, Palau

## Abstract

*Objective* Gestational diabetes mellitus (GDM) in Palau and across the Pacific Islands is a serious public health issue that is currently understudied. *Methods* This study was a retrospective cohort study that included 1730 women with a single live birth in Palau between January 2007 and December 2014. *Results* The overall prevalence of GDM among women in Palau was 5.5%. Women who were older (≥30 years) or obese (BMI ≥30) were more likely to have GDM than women who were younger (<30 years) or non-obese (BMI <30), respectively. When adverse birth outcomes were assessed, women with GDM were found to have significantly higher prevalence of high birth weight infants, cesarean sections, and neonatal deaths when compared to women without GDM. In fact, women with GDM were five times more likely to have a neonatal death than women without GDM (p = 0.008). *Conclusion* Reducing overall rates of obesity in the population could help reduce rates of diabetes and GDM in Palau. Based on this work, current practices for the identification, monitoring and treatment of women with GDM should be evaluated and strengthened in order to reduce neonatal mortality rates in Palau.

## Significance

*What is already known on this subject?* Prevalence of gestational diabetes (GDM) varies globally, but has not been well explored in the Pacific region. Factors such as maternal age and obesity are well known to influence risk of developing GDM. Women with GDM have increased risk complications to herself and her infant.

*What this study adds?* Prevalence of GDM in Palau was 5.5% between 2007 and 2014. Women who were older or obese were found to be at greater risk for gestational diabetes. Women with GDM in Palau were found to have greater risk of macrosomia, cesarean section, and neonatal mortality.

## Introduction

Gestational diabetes mellitus (GDM) is defined as glucose intolerance of variable degree with onset or first recognition during pregnancy. GDM can cause prenatal and maternal complications for women such as: preeclampsia, cesarean delivery, and an increased risk of developing type 2 diabetes later in life. Children born to women with GDM have increased risk of developing macrosomia, childhood obesity, pre-diabetes, and type 2 diabetes (American Diabetes Association 2014; Wendland et al. 2012).

The Centers for Disease Control and Prevention (CDC) reported that 2–10% of pregnant women in the U.S. have gestational diabetes and that immediately after pregnancy 5–10% were found to have type 2 diabetes. Additionally, those women who had gestational diabetes have a 35–60% chance of developing diabetes in the next 10–20 years (Moyer 2014; Nanditha et al. 2016; Oklahoma State Department of Health 2012).

Pacific Island jurisdictions have some of the highest non-communicable disease (NCD) rates in the world, including diabetes and obesity. Prevalence of NCDs also appears to be increasing among the U.S.-Affiliated Pacific Islands (USAPIs), which includes Palau as well as American Samoa, Commonwealth of the Northern Mariana Islands, Federated States of Micronesia, Guam, and Republic of the Marshall Islands (Wasem 2004; World Bank 2014). This is due to modernization in these islands and associated nutritional transition (Charlton et al. 2016).

Palau is an independent nation located in the Western Pacific between Guam and the Philippines with an estimated population of 17,501 in 2012 (Budget & Planning Office, Republic of Palau 2012). Palau consists of more than 340 islands, though only eight are inhabited. Today, approximately 70% of the population reside in Koror and the remaining 30% scattered throughout the islands of Babeldaob and the outlying states of Kayangel, Peleliu, Angaur, Sonsorol, Pulo Anna, Hatohobei and Helen’s Reef. The majority of the population in Palau are ethnically Palauan.

There is only one hospital facility in Palau located in Koror. Prenatal care is provided to all pregnant women in Palau for free at the Community Health Center. About 99.9% of births are delivered in the hospital(Republic of Palau 2015). The total fertility rate in Palau is 2.21 according to most recent statistics (Republic of Palau 2015).

According to the 2011 STEPS survey report, the estimated prevalence of diabetes in Palau among adults aged 25–64 years of age was 36.5%. Additionally, 42.2% of women in the age group of 25–34 had a body mass index (BMI) of >=30 (95% CI 35.9–48.5) and 46.3% (95% CI 41.7–50.9) for ages 35–44 (Ministry of Health, Republic of Palau 2013).

In May 2010, the Pacific Island Health Officers Association declared a state of health emergency due to the epidemic of chronic diseases in the USAPIs based on available data in the region (Pacific Island Health Officers Association 2010; Ichiho et al. 2013). To respond to this emergency, there has been an active effort to improve chronic disease surveillance systems to monitor NCD trends among the USAPIs by strengthening data collection systems and building epidemiological capacity. Even though NCD surveillance has improved in Palau, overall research and surveillance of sexual and reproductive health in the region is weak, and the prevalence of gestational diabetes in Palau and the rest of the USAPIs remains unknown.

The aims of this study were to establish the prevalence of GDM in Palau, identify determinants of GDM in Palau, and assess the associations of GDM with selected birth outcomes in Palau.

## Methods

### Study Population/Participants

All pregnant women who delivered a live singleton birth at the Belau National Hospital from 2007 to 2014 were included in this study.

### Data Collection/Measures/Exclusions

This study was a retrospective cohort study of women who gave birth in Palau between January 2007 and December 2014. The source of data included the 2007 to 2014 birth registry records and birth certificates for birth data: demographics, prenatal and maternal outcomes. The chronic diseases electronic management system (CDEMS) was used to identify women with pre-existing diabetes. Data was linked to the CDEMS database using the woman’s patient ID and verified by name and date of birth.

Data were available for 2138 singleton deliveries from 2007 to 2014 before exclusions. Women who were diagnosed with pre-existing diabetes (n = 48), had no documented blood and glucose results and those that did not take the 2nd stage OGTT test (n = 236), had a non-live birth (n = 19), and those women who never came to antenatal care (ANC) during their pregnancy (n = 105) were excluded from this study. Additionally, 438 women with multiple deliveries between 2007 and 2014 were considered and only the most recent birth was kept which excluded an additional 236 deliveries. Therefore the final sample size included 1730 mother–child pairs.

GDM screening in Palau follows the two-stage approach using of 1 h 50-g oral glucose challenge test (OGCT) and second stage of 3 h oral glucose tolerance test (OGTT) between 24 and 28 weeks of gestation as recommended by the U.S. Preventive Services Task Force (Hillier 2008; Moyer 2014; Metzger 2011).

Data variables used in analysis include: Patient hospital ID, woman’s age at delivery, race/ethnicity (Palauan vs. other), pre-pregnancy BMI, glucose test result, delivery date, number of ANC visits, tobacco use, type of delivery (normal spontaneous vaginal delivery or cesarean section) and gestational age. Infant outcomes include: birth weight, weeks of gestation, and neonatal status (live births and infant deaths of <28 days). Variables for analysis were selected based on known risk factors as well as availability of data in Palau.

### Statistical Analysis

Data were analyzed using SPSS 21. The chi square test for differences in proportions (categorical variables) was used. A p value of <0.05 was considered to be statistically significant. Univariate and multivariate logistic regression was performed to determine predictors of the selected outcomes. Best-fit practices were used and adjusted odds-ratios were estimated using a multiple logistic regression model. Independent variables with no association to the dependent variable were excluded from multivariate models.

### Ethics Approval

This study was a product of the Operational Research Course, which was funded by the International Union Against Tuberculosis and Lung Disease (The Union) and The Pacific Community (SPC), therefore permission for the study was sought from the International Union Against Tuberculosis and Lung Disease and local Ethics approval from Palau Institutional Review Board.

## Results

A total of 1730 women who gave birth or 88% of the total deliveries between 2007 and 2014 were included in this study. Annual cases of GDM ranged from 4 to 20 from 2007 to 2014, and overall, 5.5% of women in this study were diagnosed with GDM (Table 1).

**Table 1:** Annual prevalence of gestational diabetes (GDM) in Palau, 2007–2014

Year	Total number of deliveries	GDM cases N (%)
2007	54	4 (1.4)
2008	221	17 (5.8)
2009	268	8 (2.9)
2010	241	18 (7.3)
2011	237	20 (8.2)
2012	254	6 (2.3)
2013	220	7 (3.1)
2014	235	15 (6.3)
Total	1730	95 (5.5)

Women with GDM were significantly more likely to be older (≥30 years), and obese (BMI ≥30) prior to pregnancy than women without GDM (Table 2). In comparing adverse birth outcomes by GDM status, women with GDM were significantly more likely to have a high birth weight infant, deliver by cesarean section, and have a neonatal death. (Table 3).

**Table 2:** Demographic characteristics among women in Palau by GDM status, 2007–2014

	GDM N (%)	Non-GDM N (%)	*P* value
Overall	95 (5.5)	1635 (94.5)	
Age			
<20	3 (3.2)	179 (10.9)	<**0.001**
20–29	27 (28.4)	729 (44.6)	
≥30	65 (68.4)	727 (44.5)	
Race/ethnicity			
Palauan	81 (85.3)	1368 (83.7)	0.914
Filipino	10 (10.5)	187 (11.4)	
Other	4 (4.2)	80 (4.9)	
Pre-pregnancy BMI			
Underweight (<18.5)	3 (3.2)	88 (5.4)	<**0.001**
Normal (18.5–24.9)	22 (23.2)	708 (43.3)	
Overweight (25.0–29.9)	27 (28.4)	431 (26.4)	
Obese (30.0+)	43 (45.3)	408 (25.0)	
Tobacco use			
Smoke	0 (0.0)	7 (0.4)	0.870
Chew	64 (67.4)	1059 (64.8)	
Non-user	30 (31.6)	544 (33.3)	

Two women were missing tobacco use data

Bold values are significant at the alpha = 0.05 level

**Table 3:** Birth outcomes of babies born to women in Palau, by GDM status, 2007–2014

Birth outcomes	GDM N (%)	Non-GDM N (%)	*P* value
Birthweight			
Low (<2500 g)	11 (11.7)	151 (9.2)	**0.002**
Normal (2500–4000 g)	72 (76.6)	1416 (86.7)	
High (>4000 g)	11 (11.7)	67 (4.1)	
Missing	2		
Term			
Pre-term (<37 weeks)	27 (28.4)	351 (21.5)	0.112
Full-term (≥37 weeks)	68 (71.6)	1283 (78.5)	
Missing	2		
Neonatal status			
Alive at 28 days	91 (95.8)	1621 (99.1)	**0.002**
Neonatal deaths (death <28 days)	4 (4.2)	14 (0.9)	
Delivery type			
Normal Spontaneous Vaginal Delivery	43 (45.3)	1091 (66.7)	<**0.001**
Cesarean Section	52 (54.7)	544 (33.3)	

Two infants were missing birthweight and term data

Bold values are significant at the alpha = 0.05 level

In a multivariate model, women ≥30 years old were found to be 3.8 times more likely to develop GDM than younger women (<20 years old) (Table 4). In this same model, women obese prior to pregnancy (BMI ≥30) were 2.7 times more likely to develop GDM than women with a healthy pre-pregnancy BMI (<25) (Table 4). Race was not found to be significantly associated with GDM in Palau.

**Table 4:** Crude and adjusted odds ratios for GDM in Palau, 2007–2014

	Crude OR (95% CI)	*P* value	Adjusted OR (95% CI)	*P* value
Age				
<20	Ref		Ref	
20–29	2.21 (0.66–7.4)	0.197	1.85 (0.55–6.23)	0.320
≥30	5.34 (1.66–17.17)	**0.005**	3.80 (1.15–12.58)	**0.029**
BMI				
<25	Ref		Ref	
25 to <30	1.99 (1.14–3.48)	**0.015**	1.69 (0.94–3.04)	0.080
30+	3.36 (2.02–5.57)	< **0.001**	2.68 (1.54–4.68)	**0.001**
Race/ethnicity				
Other	Ref		Ref	
Palauan	1.13 (0.63–2.02)	0.682	0.96 (0.51–1.81)	0.898

Bold values are significant at the alpha = 0.05 level

In a multivariate model examining risk factors for neonatal death, women with GDM were found to be 4.9 times more likely to have a neonatal death than women without GDM when age, BMI, and tobacco use were controlled for (Table 5).

**Table 5:** Crude and adjusted odds ratios for neonatal mortality (<28 days) in Palau, 2007–2014

	Crude OR (95% CI)	*P* value	Adjusted OR (95% CI)	*P* value
Age				
<20	Ref		Ref	
20–29	0.84 (0.17–4.08)	0.830	0.73 (0.15–3.61)	0.694
≥30	1.03 (0.22–4.83)	0.966	0.75 (0.15–3.82)	0.730
BMI				
<25	Ref		Ref	
25 to <30	1.28 (0.41–4.07)	0.671	1.15 (0.35–3.84)	0.817
30+	1.57 (0.52–4.69)	0.421	1.25 (0.38–4.13)	0.713
Tobacco use				
No	Ref		Ref	
Yes	1.39 (0.49–3.90)	0.54	1.33 (0.46–3.86)	0.606
GDM				
No	Ref		Ref	
Yes	5.09 (1.64–15.77)	**0.005**	4.93 (1.53–15.89)	**0.008**

Bold values are significant at the alpha = 0.05 level

## Discussion

### Comparison with Other Studies

Overall, the GDM prevalence in Palau was determined to be 5.5% from 2007 to 2014. The International Diabetes Funds states that GDM develops in 4% of all pregnancies worldwide, so Palau appears to have a relatively high prevalence of GDM (International Diabetes Federation 2015). This suggests that GDM should be further monitored and investigated in Palau, as well as the rest of the Pacific.

In general, older women and pre-pregnancy obesity were both significant independent predictors associated with GDM. These findings are consistent with previous findings in other populations (Chang et al. 2015; Lawrence et al. 2007; Nanditha et al. 2016). However, this work is the first time that this relationship has been established in Palau or other Pacific Island Jurisdictions to the best of our knowledge.

In this study we found that GDM is associated with higher rates of high birth weight, cesarean section, and neonatal death. Strikingly, women in Palau with gestational diabetes were five times more likely to have a neonatal death than women without GDM, although we cannot prove causation from this study alone. Some previous work has demonstrated an association between GDM and neonatal mortality, though results vary from study to study (Billionnet et al. 2017). It should be noted that most of this work has been conducted in developed nations where prenatal care, and diagnosis and treatment of GDM is much more advanced than in Palau. Previous work has shown the hyperglycemia during pregnancy can increase risk of perinatal mortality in less developed nations (Duncan et al. 2011). So, it is possible that women with GDM in Palau have poorer control of GDM than women in more developed nations, thus resulting in increased risk of neonatal mortality.

This work strongly highlights the need to not only screen for gestational diabetes during pregnancy but to closely monitor those pregnant women diagnosed with GDM. Previous studies have shown that if women with gestational diabetes are properly managed, risk of neonatal death can be reduced (Buchanan and Xiang 2005; Freeman et al. 2015; Kim et al. 2007; Balaji et al. 2014).

### Strengths and Limitations

One strength of this study is that it includes all pregnant women who gave birth between 2007 and 2014, and data on relevant risk factors was mostly complete. Additionally, the majority of these women (88%) received a test for gestational diabetes according to the recommended guidelines by U.S. Preventive Services Task Force (Hillier 2008; Moyer 2014). Also, women with pre-existing DM were identified and confirmed and excluded from this study. This study adhered to STROBE Guidelines.

A limitation is that still birth, congenital anomalies, and other birth complication data were not routinely collected in the birth registry, therefore these indicators were not assessed in this study. In 2007, data on blood sugar level were not routinely collected therefore the majority of the data were excluded from this year resulting in a seemingly low number of deliveries and GDM cases. As with most small populations, there is high stochasticity of GDM rates from year to year. Additionally, we were not able to evaluate treatment of women diagnosed with GDM to assess control of disease. Finally, we were not able to follow up women diagnosed with GDM to determine whether or not they had DM post-pregnancy to differentiate between undiagnosed DM prior to pregnancy and GDM.

## Conclusion

Overall, promotion of and support for healthy lifestyles in Palau, especially among pregnant women is essential. Reducing overall rates of obesity in the population could potentially help reduce risk of diabetes and gestational diabetes. Additionally, there is no coordinated effort to track women with GDM or those women who are at risk of developing the disease. Development of evidence-based, culturally-appropriate interventions for weight loss and management for women with gestational diabetes is highly recommended. By reducing rates of GDM and proper management of pregnant women with GDM in Palau we can potentially reduce out neonatal mortality rates and improve the overall health of the population of Palau.

## References

[CR2] American Diabetes Association (2014). Standards of medical care in diabetes. Diabetes Care.

[CR3] Balaji, V., Balaji, M. S., Rajendran, R., Nielsen, K. K., Radhakrishnan, R., Kapur, A., & Seshiah, V. (2014). A cohort study of gestational diabetes mellitus and complimentary qualitative research: Background, aims and design. *BMC Pregnancy and Childbirth*, 14:378. Retrieved from http://www.biomedcentral.com/1471-2393/14/378. 10.1186/s12884-014-0378-y PMC4248438 25421525

[CR4] Billionnet C, Mitanchez D, Weill A, Nizard J, Alla F, Hartemann A, Jacqueminet S (2017). Gestational diabetes and adverse perinatal outcomes from 716,152 births in France in 2012. Diabetologia.

[CR5] Buchanan TA, Xiang AH (2005). Gestational diabetes mellitus. The Journal of Clinical Investigation.

[CR21] Budget & Planning Office, Republic of Palau. (2012). *Ministry of Finance*. Retrieved from Palau Government: http://palaugov.pw/executive-branch/ministries/finance/budgetandplanning/population-census/.

[CR6] Chang A, Hurwitz E, Miyamura J, Kaneshiro B, Sentell T (2015). Maternal risk factors and perinatal outcomes among pacific islander groups in Hawaii: A retrospective cohort study using statewide hospital data. BMC Pregnancy Childbirth.

[CR7] Charlton KE, Russell J, Gorman E, Hanich Q, Delisle A, CampBell B, Bell J (2016). Fish, food security and health in Pacific Island countries and territories: a systematic literature review. BMC Public Health.

[CR8] Duncan BB, Mengue SS, Schmidt MI (2011). Lesser than diabetes hyperglycemia in pregnancy is related to perinatal mortality: A cohort study in Brazil. BMC Pregnancy and Childbirth.

[CR9] Freeman J, Thompson K, Muasau-Howard B, Ah Ching J, McGarvey S, Hawley N (2015). An evaluation of gestational diabetes mellitus screening practices in American Samoa. Pacific Journal of Reproductive Health.

[CR10] Hillier T (2008). Screening for gestational diabetes mellitus: A systematic review for the U.S. preventive services task force. Annals of Internal Medicine.

[CR11] Ichiho HM, Demei Y, Kuartei S, Aitaoto N (2013). An assessment of non-communicable diseases, diabetes, and related risk factors in the Republic of Palau: A system perspective. Hawaii Journal of Medicine & Public Health.

[CR12] International Diabetes Federation. (2015). *Unite for Diabetes*. Retrieved from GDM Resources: http://www.idf.org/women-and-diabetes/resource-centre.

[CR13] Kim C, McEwen L, Piette J, Goewey J, Ferrara A, Walker E (2007). Risk Perception for diabetes among women with histories of gestational diabetes mellitus. Diabetes Care.

[CR14] Lawrence J, Contreras R, Chen W, Sacks D (2007). Trends in the prevelance of preexisting diabetes and gestational diabetes mellitus among racially/ethnically diverse population of pregnant women. Diabetes Care.

[CR15] Metzger, B. (2011, August 9). *Detecting and Diagnosing Gestational Diabetes*. Retrieved from Physician’s Weekly: http://www.physiciansweekly.com/Features/11_30/gestational_diabetes.html.

[CR16] Ministry of Health, Republic of Palau. (2013). *Palau NCD STEPS Report*. Palau. Retrieved from Palau Health: http://www.palauhealth.org/files/PALAU%20NCD%20STEPS%20REPORT.PDF.

[CR17] Moyer V (2014). Screening for gestational diabetes mellitus. Annals of Internal Medicine.

[CR18] Nanditha, A., Ma, R., Ramachandran, A., Snehalatha, C., Chan, J., & Chia, K. (2016). Diabetes in Asia and the Pacific: Implications of the global epidemic. *Diabetes Care*, 472–485. 10.2337/dc15-1536 26908931

[CR19] Oklahoma State Department of Health. (2012). Gestational Diabetes Among Oklahoma Mothers. *Oklahoma Pregnancy Risk Assessment Monitoring System*.

[CR20] Pacific Island Health Officers Association. (2010). *PIHOA NCD Declaration*. PIHOA.

[CR1] Republic of Palau. (2015). *Budget & Planning Office*. Retrieved from ROP Statistical Yearbooks: http://palaugov.pw/rop-statistical-yearbooks/.

[CR22] Wasem, C. (2004). *U.S. Affiliated Pacific Basin Jurisdictions: Legal, Geographic and Demographic Information*. Retrieved from Rural Health Information Hub: http://www.ruralhealthinfo.org/resources/2065.

[CR23] Wendland E, Torloni M, Falavigna M, Trujillo J, Dode M, Campos M (2012). Gestational diabetes and pregnancy outcomes-a systematic review of world health organization and the international association of diabetes in pregnancy study groups diagnostic criteria. BMC Pregnancy Childbirth.

[CR24] World Bank. (2014). *Non-Communicable Disease (NCD) Roadmap Report*. Retrieved from World Bank: http://www.documents/worldbank.org/curated/en/2014/19778739/communicable-disease-ncd-roadmap-report.

